# Supplementary material for: Rapid on-site universal vertebrate species identification via multi-barcode nanopore sequencing
Source: PLoS One. 2025 Nov 11;20(11):e0336383. doi: 10.1371/journal.pone.0336383 (PMC12604797; doi:10.1371/journal.pone.0336383)
Supplement: S1 Fig — a) Schematic diagram of typical vertebrate mtDNA, with approximate positions of the four barcode sequences. Primer sequences are given in S2 Table in S1 File. b) Schematic representation of the relationship between the primers in this study and previously published primers. Numbers indicate coordinates, based on the human mtDNA reference sequence (NC_012920). c) Detailed comparison of primer sequences for barcodes COI, CYTB, and 12S rRNA. 16S rRNA primers are not shown since they have no overlaps with previously described primers. (PDF) [file pone.0336383.s001.pdf]

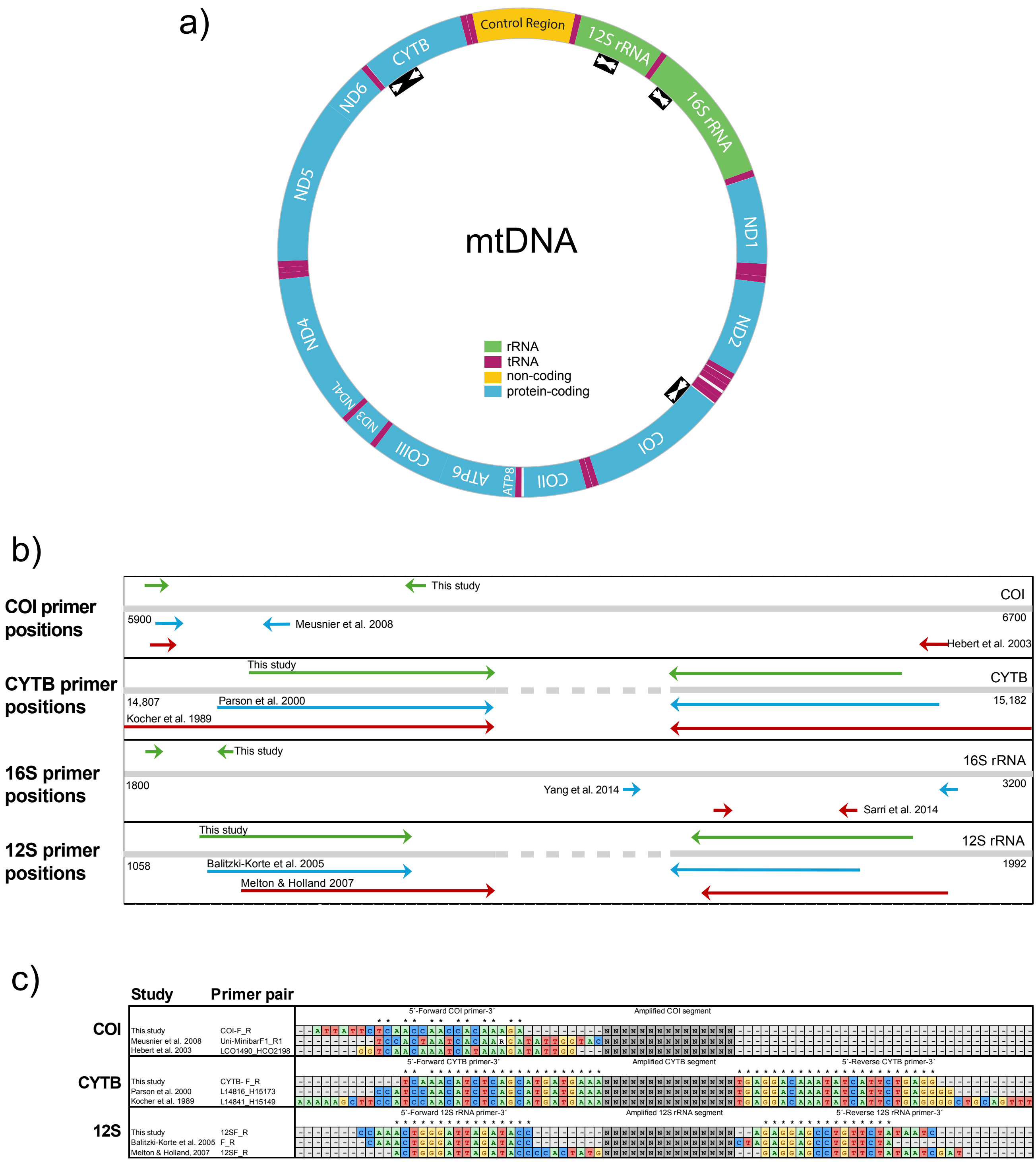

S1 Fig: Barcode primer positions in mtDNA, and relationships to previously designed primers.

a) Schematic diagram of typical vertebrate mtDNA, with approximate positions of the four barcode sequences. Primer sequences are given in S2 Table.
b) Schematic representation of the relationship between the primers in this study and previously published primers. Numbers indicate coordinates, based on the human mtDNA reference sequence (NC_012920).
c) Detailed comparison of primer sequences for barcodes COI, CYTB, and 12S rRNA. 16S rRNA primers are not shown since they have no overlaps with previously described primers.
