# Supplementary material for: Rapid on-site universal vertebrate species identification via multi-barcode nanopore sequencing
Source: PLoS One. 2025 Nov 11;20(11):e0336383. doi: 10.1371/journal.pone.0336383 (PMC12604797; doi:10.1371/journal.pone.0336383)
Supplement: S5 Fig — a) A NJ-K2P tree with 100 bootstrap replicates was calculated using MEGA (Hall, 2013) and displayed using the interactive Tree Of Life (Letunic and Bork, 2021) for the chosen 103-bp segment of 16S rRNA. Clades have been collapsed when multiple sequences of the same species (or closely related species) were present. Instances where species differentiation was not possible are displayed in panels b) to h). Inconsistent species identifications are highlighted by red underlining. (PDF) [file pone.0336383.s005.pdf]

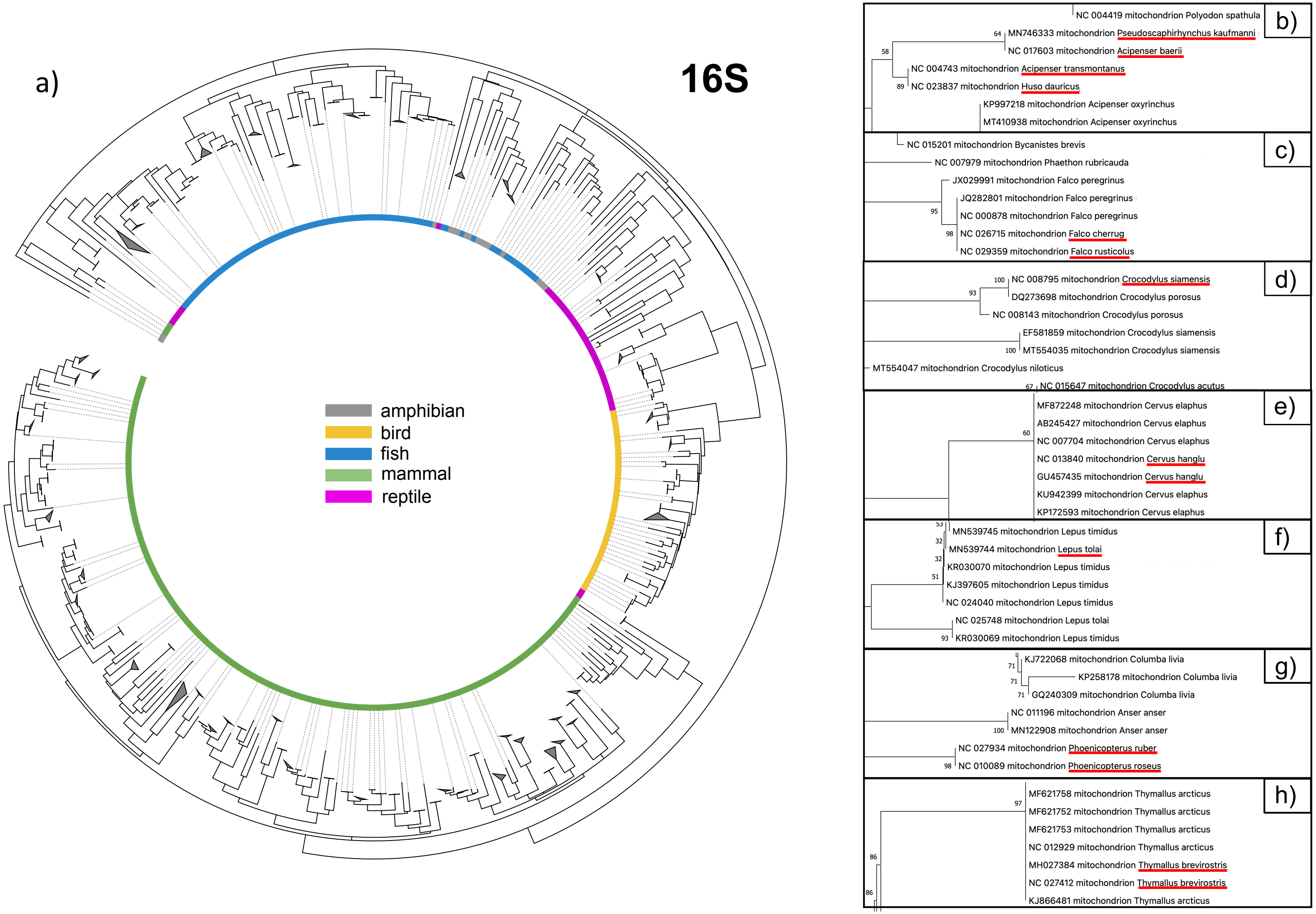

S5 Fig: Phylogenetic tree based on 16S ribosomal RNA.

a) A NJ-K2P tree with 100 bootstrap replicates was calculated using MEGA (Hall, 2013) and displayed using the interactive Tree Of Life (Letunic and Bork, 2021) for the chosen 103-bp segment of 16S rRNA. Clades have been collapsed when multiple sequences of the same species (or closely related species) were present. Instances where species differentiation was not possible are displayed in panels b) to h). Inconsistent species identifications are highlighted by red underlining.
