# Supplementary material for: Rapid on-site universal vertebrate species identification via multi-barcode nanopore sequencing
Source: PLoS One. 2025 Nov 11;20(11):e0336383. doi: 10.1371/journal.pone.0336383 (PMC12604797; doi:10.1371/journal.pone.0336383)
Supplement: S6 Fig — a) PCR products are generated by primers with 26-nt 5´ extensions to increase the target size for transposome complex binding. b) Transposome complex introduces a double-strand break at a random point within each duplex and adds a sample-identifying index to each of the two internal free 5´ ends. c) Following sequencing adapter addition, sequencing proceeds from each adapter through the nanopore in the 5´-3´ direction. d) In bioinformatic processing, a read is only retained if it contains a primer sequence at the distal end (primer sequences are then trimmed – not shown), and reads are used as input with NGSpeciesID for consensus sequence generation. This leads to reduced confidence at the proximal end of each strand consensus. Top- and bottom-strand consensuses are used separately for database query and only results with consensus sequences based on at least 100 independent reads and generated in both directions are considered (though see main text). (PDF) [file pone.0336383.s006.pdf]

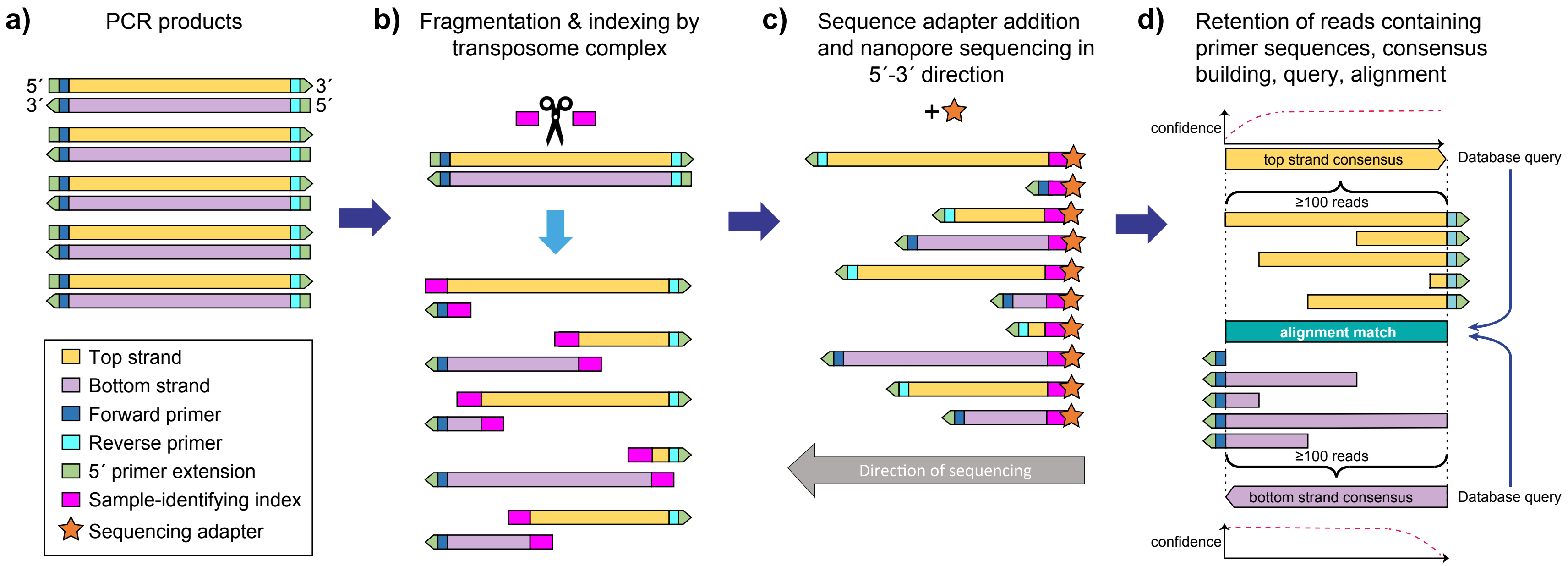

S6 Fig: Action of the rapid indexing kit and generation of sequences for database query.

a) PCR products are generated by primers with 26-nt 5´ extensions to increase the target size for transposome complex binding. **b)** Transposome complex introduces a double-stand break at a random point within each duplex and adds a sample-identifying index to each of the two internal free 5´ ends. **c)** Following sequencing adapter addition, sequencing proceeds from each adapter through the nanopore in the 5´-3´ direction. **d)** In bioinformatic processing, a read is only retained if it contains a primer sequence at the distal end (primer sequences are then trimmed - not shown), and reads are used as input with NGSpeciesID for consensus sequence generation. This leads to reduced confidence at the proximal end of each strand consensus. Top- and bottom-strand consensuses are used separately for database query and only results with consensus sequences based on at least 100 independent reads and generated in both directions are considered (though see main text).
