# Supplementary material for: Rapid on-site universal vertebrate species identification via multi-barcode nanopore sequencing
Source: PLoS One. 2025 Nov 11;20(11):e0336383. doi: 10.1371/journal.pone.0336383 (PMC12604797; doi:10.1371/journal.pone.0336383)
Supplement: S7 Fig — For each mixture, the first column indicates the relative proportion of reads expected for each species present in the mixture for that indicated barcode in the minibarcode tetraplex. The second column for each mixture indicates the relative proportion of reads assigned to the barcode of each of the detected species based on the highest number of supporting reads of that species’ barcode consensus sequence. In sequence processing consensus sequences with >20 supporting reads were retained. A species was only considered present if forward and reverse consensus sequences were generated for at least two species ID barcodes. (PDF) [file pone.0336383.s007.pdf]

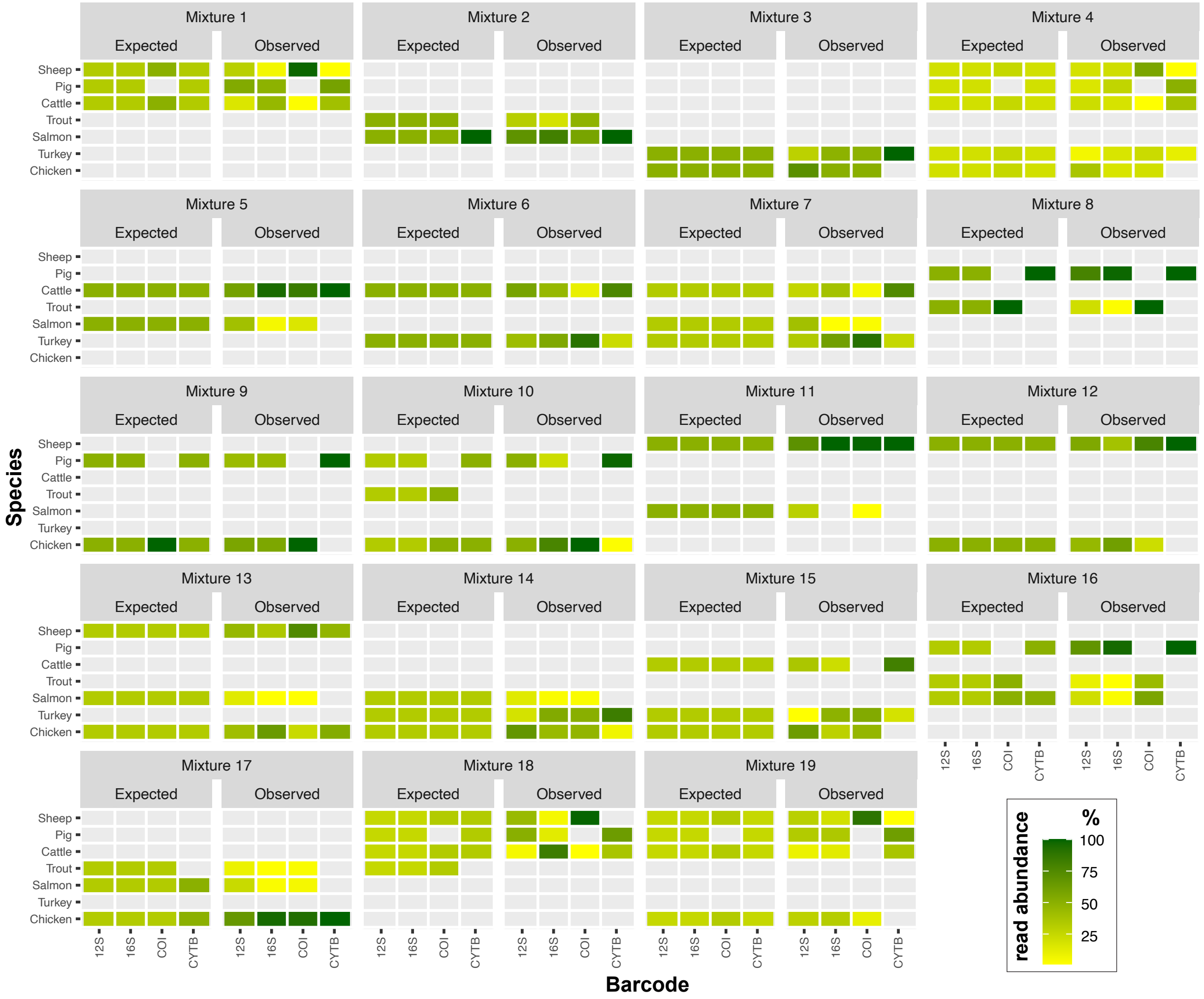

S7 Fig: Heatmap displaying the relative percentage of reads assigned to a given species within 19 lab-prepared mixtures.

For each mixture, the first column indicates the relative proportion of reads expected for each species present in the mixture for that indicated barcode in the minibarcode tetraplex. The second column for each mixture indicates the relative proportion of reads assigned to the barcode of each of the detected species based on the highest number of supporting reads of that species' barcode consensus sequence. In sequence processing consensus sequences with >20 supporting reads were retained. A species was only considered present if forward and reverse consensus sequences were generated for at least two species ID barcodes.
